# A behaviour change package to prevent hand dermatitis in nurses working in the National Health Service: results of a cluster randomized controlled trial†

**DOI:** 10.1111/bjd.18862

**Published:** 2020-03-10

**Authors:** I. Madan, V. Parsons, G. Ntani, D. Coggon, A. Wright, J. English, P. McCrone, J. Smedley, L. Rushton, C. Murphy, B. Cookson, H.C. Williams

**Affiliations:** ^1^ Occupational Health Service Guy's and St Thomas’ NHS Foundation Trust and King's College London London U.K.; ^2^ MRC Lifecourse Epidemiology Unit University of Southampton Southampton U.K.; ^3^ Centre for Behaviour Change, Department of Clinical, Educational and Health Psychology University College London London U.K.; ^4^ Dermatology Circle Nottingham NHS Treatment Centre Nottingham U.K.; ^5^ Centre for the Economics of Mental and Physical Health King's College London London U.K.; ^6^ Occupational Health Service University Hospital Southampton NHS Foundation Trust Southampton U.K.; ^7^ Epidemiology and Biostatistics Imperial College London London U.K.; ^8^ King's Clinical Trial Unit King's College London London U.K.; ^9^ Medical Microbiology University College London London U.K.; ^10^ Centre of Evidence Based Dermatology University of Nottingham Nottingham U.K.

## Abstract

**Background:**

Occupational hand dermatitis poses a serious risk for nurses.

**Objectives:**

To evaluate the clinical and cost‐effectiveness of a complex intervention in reducing the prevalence of hand dermatitis in nurses

**Methods:**

This was a cluster randomized controlled trial conducted at 35 hospital trusts, health boards or universities in the UK. Participants were (i) first‐year student nurses with a history of atopic conditions or (ii) intensive care unit (ICU) nurses. Participants at intervention sites received access to a behavioural change programme plus moisturizing creams. Participants at control sites received usual care. The primary outcome was the change of prevalent dermatitis at follow‐up (adjusted for baseline dermatitis) in the intervention vs. the control group. Randomization was blinded to everyone bar the trials unit to ensure allocation concealment. The trial was registered on the ISRCTN registry: ISRCTN53303171.

**Results:**

Fourteen sites were allocated to the intervention arm and 21 to the control arm. In total 2040 (69·5%) nurses consented to participate and were included in the intention‐to‐treat analysis. The baseline questionnaire was completed by 1727 (84·7%) participants. Overall, 789 (91·6%) ICU nurses and 938 (84·0%) student nurses returned completed questionnaires. Of these, 994 (57·6%) had photographs taken at baseline and follow‐up (12–15 months). When adjusted for baseline prevalence of dermatitis and follow‐up interval, the odds ratios (95% confidence intervals) for hand dermatitis at follow‐up in the intervention group relative to the controls were 0·72 (0·33–1·55) and 0·62 (0·35–1·10) for student and ICU nurses, respectively. No harms were reported.

**Conclusions:**

There was insufficient evidence to conclude whether our intervention was effective in reducing hand dermatitis in our populations.

**Linked Comment:** Brans. *Br J Dermatol* 2020; 183:411–412.

## Abstract 2

**What is already known about this topics?**

- Nurses are at high risk of developing hand dermatitis.
- Educational interventions are only partially successful in preventing hand dermatitis in healthcare workers.

**What does this study add?**

- The nurse participants in our study had a high level of positive beliefs about good hand care; however, this was not translated sufficiently into their behaviours even when they were provided with access to a behavioural change programme and given ready access to hand moisturizers.
- The behavioural change programme intervention did not have a significant effect on reducing hand dermatitis among at‐risk nurses.

**Linked Comment:** Brans. *Br J Dermatol* 2020; 183:411–412.

Plain language summary available online

Occupational hand dermatitis is a major hazard in nurses (point prevalence 18–30%).^1^ It often develops in the first few years after joining the profession,^1^, ^2^ it impairs quality of life and it may lead to job loss.^3^, ^4^ The prognosis for established hand dermatitis is poor.^5^ The high prevalence of irritant hand dermatitis in nurses is associated with frequent hand washing and wearing occlusive gloves. Nurses may develop allergic contact dermatitis caused by exposure to sensitizers (e.g. rubber components) in the workplace.^6^ UK practice guidelines^7^ recommend regular application of emollients for prevention, although use of moisturizing creams by nurses is low.^8^, ^9^ Antibacterial hand rubs rather than hand washing with soap are recommended for hand cleansing when the hands are not visibly contaminated by body fluids.^10^ Correct hand drying with paper towels after washing is essential.^11^

Evidence that hand dermatitis in nurses is reduced by interventions that incorporate the above measures^11^, ^12^, ^13^, ^14^, ^15^, ^16^ is limited by a lack of standardized methods, small study size or failure to address cost‐effectiveness.^17^ While educational programmes and individual counselling to promote optimal hand care behaviours among healthcare workers have shown encouraging results,^14^, ^16^ their delivery in the field is challenging. Authors have called for high‐quality trials, using interventions based on psychological theory.^14^, ^16^, ^18^ In a study of patients with occupational hand dermatitis receiving inpatient tertiary prevention, variables based on the theory of planned behaviour explained 30% of the variance in postintervention behaviours to prevent dermatitis, and 38% of the variance in intentions for preventive behaviours.^19^

To address continuing uncertainty about the clinical and cost‐effectiveness of interventions designed to prevent hand dermatitis among nurses, we undertook a pragmatic trial of a behavioural change programme (BCP) aimed at improving adherence to preventive measures.

## Patients and methods

We conducted a cluster randomized controlled trial in National Health Service trusts, health boards and universities (sites) across Britain, with sites, or clusters of neighbouring sites, as the units of randomization. The trial protocol has been described elsewhere.^20^ Sites were eligible for inclusion if they had an occupational health service and either trained student nurses or had an adult or paediatric intensive care unit (ICU). They were randomly allocated to an intervention or control arm.

### Participants

There were two groups: (i) student nurses about to start their first clinical placement and who were at increased risk of hand dermatitis because of a history of atopic disease (history of eczema, hay fever or asthma) or hand dermatitis; and (ii) full‐time ICU nurses who were at increased risk of hand dermatitis through workplace exposure. Informed written consent was obtained from all participants.

### Intervention

The intervention comprised a BCP that targeted appropriate use of gloves; washing hands with soap and water only when the hands were visibly soiled, otherwise using antibacterial hand rubs;^6^ using moisturizing cream before, during and after shifts;^6^, ^21^ and contacting occupational health early if hand dermatitis occurred. The BCP was supported by provision of a personal supply of hand moisturizer to student nurses and a regular supply of moisturizing creams on the ICU wards. The local occupational health service, control‐of‐infection team, and line management reinforced the messages on skincare. Participants in both trial arms received a leaflet on hand care.

The BCP was based on the theory of planned behaviour and written in plain English.^22^ It aimed to change relevant attitudes, subjective norms, perceived behavioural control and intentions by providing written evidence‐based information about health, and social and environmental consequences of skincare behaviours coupled with illustrative pictures. Participants were asked to form implementation intentions for hand cream use and checking for dermatitis. The BCP was offered online or as hard copy (32‐page magazine). It was made available to ICU nurse participants when recruitment at their site was complete, and to student nurse participants 2 weeks before starting their first clinical attachment. Participants at control sites were managed according to established best practice (provision of a leaflet about optimal hand care).^21^

### Outcomes

The primary outcome was the change from baseline to follow‐up in the point prevalence of photographically discernible hand dermatitis, as assessed by two dermatologists using a bespoke photographic assessment method.^23^ Secondary outcomes were (i) the change in severity of hand dermatitis; (ii) days lost from sickness absence and days of modified duties because of hand dermatitis per 100 days of nurse time; (iii) change in beliefs about dermatitis prevention behaviours; (iv) change in the reported frequency of use of hand rubs for hand cleansing, hand washing with water, and use of moisturizing creams; (v) change in quality of life score (EuroQol 5 dimensions 5 levels, EQ‐5D‐5L)^24^ and (vi) the extent to which the moisturizer provided for the intervention was used.

### Sample size and statistical power

The sample size was based on the participation of 26 sites (clusters, with each site recruiting 40 students and 40 ICU nurses). The expected prevalence of hand dermatitis was 5% (baseline) and 25% (follow‐up) in student nurses and 25% (baseline) and 23% (follow‐up) in ICU nurses. We estimated an intraclass correlation coefficient (ICC) of 0·05 and that 20% of participants would be lost to follow‐up. With these assumptions and a 5% level of statistical significance (two sided) the study would have approximately 89% power to detect a reduction in prevalence at follow‐up in the intervention‐plus trusts to 10% in students nurses, and 95% power to detect a reduction in prevalence to 10% at follow‐up in ICU nurses.

### Randomization

Randomization (by King's Clinical Trials Unit) was carried out in four blocks as a single step at the beginning of the study. The blocks were defined according to whether centres planned to recruit students, ICU nurses or both, and centre size. Allocation was confined to the trials unit until participants were recruited and completed the baseline questionnaire. For the intention‐to‐treat analysis, the date of entry into the study was the date of consent form signature. Intervention allocation was concealed from field workers until all of the participants at that site had been recruited. The trial statistician, methodologist, dermatologists and health economist remained blinded until after the primary analysis. All nurses who consented to participate were included in the intention‐to‐treat analysis.

### Data collection

Outcome data were collected through questionnaires and standardized hand photographs^23^ at baseline and at follow‐up (ideally 12–15 months later). Participants who were unable to attend a clinic for hand photography were asked to submit hand images using their mobile phones. Intervention‐site participants were sent an ‘intermediate’ questionnaire after 3 months, which asked whether they had accessed the BCP and, if not, their reasons for not doing so.

### Statistical analysis

Statistical analysis was carried out with Stata version 12·1.^25^ The primary analysis compared changes in photographically assessed dermatitis between the two study arms according to intention to treat, and was run separately for student and ICU nurses. It used logistic regression modelling (restricted to participants with complete data on all relevant variables), with hand dermatitis at follow‐up as the outcome variable, and adjustment for the presence or absence of hand dermatitis at baseline. Final effect estimates adjusted also for follow‐up interval (treated as a continuous variable). Effect estimates were summarized by odds ratios and 95% confidence intervals of the intervention vs. control groups.

Secondary analyses explored effects on health beliefs and preventive behaviours. Health beliefs were characterized by 25 variables (treated as continuous measures), with scores ranging from 1 to 5. The effects of the intervention were assessed using separate linear regression models, with each of the 25 measures at follow‐up as outcome variables and adjustment for the corresponding measure at baseline together with the follow‐up interval. Preventive behaviours were quantified by ordinal variables, with scores for hand washing with soap and water, and use of hand rubs ranging from 1 to 4, and those for use of moisturizers from 1 to 6. In ICU nurses, the effects of the intervention on each behaviour were assessed by ordinal logistic regression, with the score at follow‐up as the outcome variable and adjustment for the corresponding score at baseline together with the follow‐up interval. Student nurses had not started their first clinical placement when they entered the trial, so it was unnecessary to adjust for their behaviour at baseline.

To account for clustering by site, random intercept modelling was used in all analyses, except where the ICC was negligible (approximately equal to 0). The main analysis was supplemented by three sensitivity analyses excluding participants (i) for whom information about dermatitis was based on their own photographs; (ii) with a follow‐up time < 12 or > 15 months; and (iii) at two sites that recruited exceptionally high numbers of student nurses.

With respect to missing data, all analyses were restricted to participants with data on the relevant outcomes. The main adjusted analysis of the primary outcome (presence of dermatitis at follow‐up) was further restricted to participants with complete data on all relevant independent variables. For the health economic analysis, we did not impute for missing data and instead conducted a complete‐case analysis.

### Ethics

Approval was granted by the Health Research Authority (reference 13/LO/0981).

## Results

### Recruitment and randomization of sites

**Figure 1:**
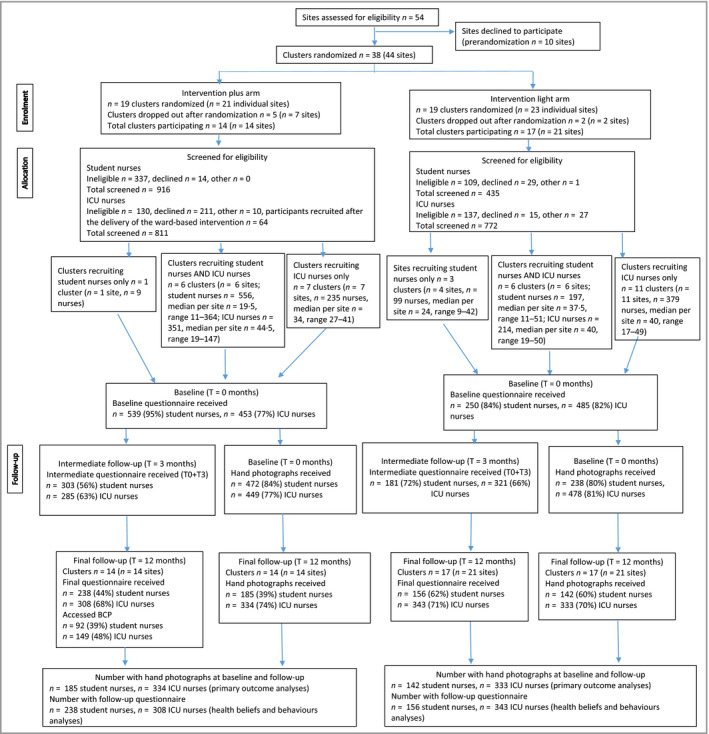
Flowchart of participants at the different stages of the study. BCP, behavioural change programme; ICU, intensive care unit.

Among 54 eligible sites, 10 declined to participate. The remaining 44 were assigned to 38 clusters comprising one (33 clusters), two (four clusters) or three (one cluster) sites. The rationale was that geographical proximity risked ‘contamination’ if students had placements at more than one site. Nineteen clusters were randomized to the intervention and 19 to the control arm. After randomization (but before recruitment of nurses), nine sites (seven clusters) withdrew due to workload concerns. The final number of 31 clusters (35 individual sites) was still sufficient for statistical power. This was more than the number of sites that we originally planned to recruit into the trial (*n* = 26 sites), and therefore this reduction did not negatively affect the power of the trial. Of those 31 clusters, 14 were randomized to the intervention and 17 to the control arm. The three clusters that comprised more than one site were all randomized to the control arm. Where the sites within these clusters recruited ICU nurses only or students only, the clusters were recorded twice on the consort diagram (Figure 1).

### Recruitment of individual participants

Participants were recruited between September 2015 and December 2016. The flow of participants through the study is illustrated in Figure 1. We screened 2934 nurses for eligibility; 2040 (69·5%) consented to participate and were included in the intention‐to‐treat analysis. The baseline questionnaire was completed by 1727 participants, giving an 84·7% response rate (789 student nurses, 91·6% response; and 938 ICU nurses, 84·0% response). Of those, 994 (57·6%) had both baseline and follow‐up photographs, and contributed to the primary outcome analyses. In total 1045 (60·5%) completed the follow‐up questionnaire and were included in the analyses of effects on health beliefs and behaviours. Among baseline questionnaire responders, the proportions providing hand photographs both at baseline and at follow‐up were lower in the intervention than in the control groups (52·3% and 64·6%, respectively), as were the proportions who completed questionnaires at both timepoints (55·0% and 67·9%).

### Characteristics of participants

Table 1 summarizes the baseline characteristics of participants according to the extent of their participation. Among student and ICU nurses, the demographic characteristics and the baseline prevalence of atopic history were similar between the intervention and control groups. There was no indication that the subsets of nurses who were included in the analyses of dermatitis, and health beliefs and behaviours, were unrepresentative of all participants.

**Table 1:** Characteristics of nurses according to level of participation

	Completed baseline questionnaire		Hand photographs at both baseline and follow‐up		Completed questionnaire at both baseline and follow‐up	
	Intervention	Control	Intervention	Control	Intervention	Control
Student nurses						
Number	539	250	185	142	238	156
Female, *n* (%)	510 (94·6)	233 (93·2)	175 (94·6)	134 (94·4)	227 (95·4)	147 (94·2)
Age (years), median (IQR)^a^	21 (19–26)	22 (19–29)	23 (19–28)	23 (19–30)	22 (19–27)	24 (19–29)
Atopy, *n* (%)^b^	531 (98·5)	238 (95·2)	181 (97·8)	135 (95·1)	233 (97·9)	148 (94·9)
Atopic dermatitis, *n* (%)	145 (26·9)	65 (26·0)	51 (27·6)	39 (27·5)	61 (25·6)	45 (28·8)
ICU nurses						
Number	453	485	334	333	308	343
Female, *n* (%)	388 (85·7)	410 (84·5)	285 (85·3)	278 (83·5)	266 (86·4)	286 (83·4)
Age (years), median (IQR)^a^	36 (27–45)	36 (27–45)	37 (28–45)	38 (28–46)	38 (28·5–47)	38 (28–46)
Hours worked per week, mean ± SD^c^	36·7 ± 2·6	36·7 ± 2·6	36·6 ± 2·5	36·7 ± 2·8	36·5 ± 2·5	36·7 ± 2·8
Atopy, *n* (%)	282 (62·3)	289 (59·6)	206 (61·7)	204 (61·3)	185 (60·1)	206 (60·1)
Atopic dermatitis, *n* (%)	58 (12·8)	63 (13·0)	45 (13·5)	40 (12·0)	42 (13·6)	36 (10·5)

ICU, intensive care unit; IQR, interquartile range. ^a^Data on age were missing for 10 student nurses (all in the control group) and seven ICU nurses (one intervention and six control). ^b^Although all student nurses were screened for atopy by the occupational health team, not all reported atopic symptoms on the questionnaire. ^c^Data on hours worked per week were missing for nine ICU nurses (four intervention and five control).

### Uptake of the behavioural change programme

Among the 519 nurses at the intervention sites who contributed to the analysis of effects on hand dermatitis, 383 (73·8%) completed the intermediate questionnaire, of whom 188 (49·1%) had accessed the BCP (42·8% of students, 53·2% of ICU nurses). However, we did not capture reliable data on the extent to which participants completed the BCP. The main reported reasons for not accessing the BCP were lack of time (38%) and forgetting (36%).

### Effects on hand dermatitis

Hand dermatitis was assessed at two timepoints (follow‐up interval 7–27 months, median 13·5). Among student nurses in the control group, its prevalence was 7% at baseline, which increased to 12% at follow‐up. In the intervention arm, the prevalence decreased from 15·1% at baseline to 10·3% at follow‐up. Clustering of the outcome among student nurses was low (ICC ≈ 0). Therefore, the final model fitted was single level, with adjustment for dermatitis at baseline and follow‐up interval.

Among ICU nurses, the baseline prevalence of dermatitis was 16·5% in both arms. Among controls it decreased to 13·8%, while in the intervention arm it decreased to 9·9%. For ICU nurses, we used a random intercept model to account for clustering by site. Estimated ICCs were 0·01 for the model adjusted only for baseline dermatitis, and 0·02 with additional adjustment for follow‐up interval.

When the intention‐to‐treat analysis was repeated after excluding nurses who reported that they did not access the BCP intervention (per protocol analysis), the change observed in objectively assessed dermatitis from baseline to follow‐up was more pronounced. The effects of the intervention using these models are summarized in Table 2. While all of the analyses suggested a small benefit from the intervention, none was statistically significant.

**Table 2:** Estimated effect of the intervention on photographically diagnosed hand dermatitis

	Student nurses	ICU nurses
Control		
Number assessed	142	333
Hand dermatitis at baseline, *n* (%)	10 (7·0)	55 (16·5)
Hand dermatitis at follow‐up, *n* (%)	17 (12·0)	46 (13·8)
Intervention		
Number assessed	185	334
Hand dermatitis at baseline, *n* (%)	28 (15·1)	55 (16·5)
Hand dermatitis at follow‐up, *n* (%)	19 (10·3)	33 (9·9)
Estimated effect of intervention		
Number^a^	327	667
OR (95% CI)^a^	0·67 (0·32–1·39)	0·65 (0·39–1·11)
Number^b^	320	647
OR (95% CI)^b^	0·72 (0·33–1·55)	0·62 (0·35–1·10)

CI, confidence interval; ICU, intensive care unit; OR, odds ratio. ^a^Prevalent hand dermatitis at follow‐up in the intervention vs. control group adjusted for baseline prevalence of dermatitis. ^b^Prevalent hand dermatitis at follow‐up in the intervention vs. control group adjusted for baseline prevalence of dermatitis and follow‐up interval.

### Effects on health beliefs

**Figure 2:**
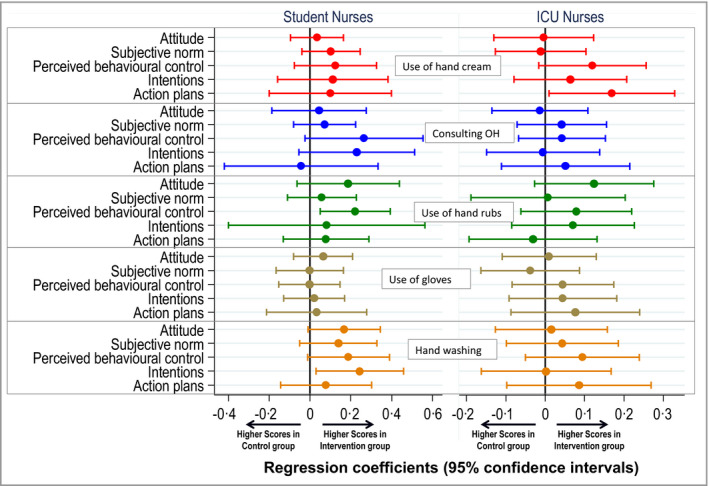
Associations between change in health belief scores from baseline to the 12‐month follow‐up and intervention group after adjusting for follow‐up time. ICU, intensive care unit; OH, occupational health.

The analyses of effects on health beliefs included 394 students and 651 ICU nurses (follow‐up interval 7·9–26·9 months, median 13·7). Figure 2 summarizes the effects of the intervention on 25 health belief scores, as estimated by random intercept or single‐level models (according to the ICC), adjusted for beliefs at baseline and follow‐up interval. The intervention had little impact on beliefs in either group, although overall there was a weak tendency for less deterioration and/or greater improvement in the intervention vs. the control arm.

### Effects on health behaviours

Differences between the intervention and control arms in the frequency of preventive behaviours at follow‐up were mostly nonsignificant when adjusted for follow‐up interval and (only in ICU nurses) for the corresponding measure assessed at baseline (Table 3). An exception was the use of moisturizers by ICU nurses, which was higher in the intervention group (adjusted odds ratio 1·59, 95% confidence interval 1·18–2·14).

**Table 3:** Estimated effect of the intervention on the frequency of preventive behaviours

Behaviour	Adjusted (in ICU nurses) for level of same behaviour at baseline^a^		Adjusted for follow‐up interval and (in ICU nurses) level of same behaviour at baseline^a^	
	Number analysed^b^	OR (95% CI)^c^	Number analysed^b^	OR (95% CI)^c^
Student nurses				
Hand washing with soap and water	392	0·81 (0·47–1·39)	384	0·83 (0·48–1·43)
Use of hand rubs	393	1·37 (0·95–1·98)	385	1·43 (0·97–2·09)
Use of moisturizing cream before shifts	394	1·14 (0·79–1·63)	386	1·22 (0·84–1·77)
Use of moisturizing cream during shifts	394	1·32 (0·79–2·21)	386	1·33 (0·91–1·92)
Use of moisturizing cream after shifts	394	1·35 (0·95–1·93)	386	1·37 (0·94–1·99)
ICU nurses				
Hand washing with soap and water	645	0·81 (0·49–1·32)	605	0·85 (0·51–1·42)
Use of hand rubs	643	1·30 (0·94–1·80)	603	1·31 (0·95–1·80)
Use of moisturizing cream before shifts	644	1·25 (0·94–1·67)	604	1·22 (0·90–1·64)
Use of moisturizing cream during shifts	644	1·70 (1·25–2·31)	604	1·59 (1·18–2·14)
Use of moisturizing cream after shifts	645	1·31 (0·90–1·92)	605	1·27 (0·88–1·85)

CI, confidence interval; ICU, intensive care unit; OR, odds ratio. ^a^No adjustment was applied for behaviours at baseline in student nurses as they had not yet started clinical work. ^b^Data on specific behaviours at baseline and/or follow‐up were missing for up to 10 student nurses and up to 48 ICU nurses. ^c^OR with 95% CI from ordinal regression. Values > 1 indicate that relative to the control group, the behaviour was more frequent at follow‐up in the intervention group.

### Effects on other outcomes

Severity of dermatitis was dichotomized (cutoff point 3).^23^ Scores ≥ 3 indicated severe dermatitis. Only two participants had severe dermatitis at baseline and three at follow‐up. As severe dermatitis was uncommon, it was not analysed further.

Severity score was defined as the average score between two dermatologists who assessed photographs. It was challenging to provide counts of participants for each category of severity (almost clear, mild, moderate). For example, 14 participants scored 2·5. For these 14, one dermatologist assessed severity as mild and the other dermatologist as moderate. Similarly, 62 participants scored 1·5, but one dermatologist assessed them as almost clear and the other as mild.

There was no difference in quality‐of‐life scores between the trial arms at baseline or follow‐up, nor for quality‐adjusted life‐years during follow‐up.

Sick leave for dermatitis was reported by five student and four ICU nurses in the intervention arm and four student and five ICU nurses in the control arm. For logistical reasons, we did not collect reliable data on supplies of moisturizers. The mean intervention costs were £14 for students and £13 for ICU nurses.

### Sensitivity analyses

After excluding data from photographs provided by the patients, the pattern of changes in prevalence of hand dermatitis was similar to that in the main analysis. None of the differences between intervention and control was statistically significant.

Restricting to participants who were followed up at 12–15 months, the prevalence of dermatitis among student nurses increased from baseline to follow‐up in both arms of the study. Among ICU nurses, it decreased in both arms. In fully adjusted models, no differences between the intervention and control groups reached statistical significance.

After excluding two trusts (both intervention sites) with high recruitment of student nurses, the prevalence of dermatitis among students increased from baseline to follow‐up in the control arm, and decreased in the intervention arm. Repeat of the main analyses with adjustment also for sex and age had no material impact on the effect estimates.

### Harms

No adverse events were reported during the study.

## Discussion

This trial found no clear benefit from a BCP in reducing the prevalence of hand dermatitis among student or ICU nurses. There was no significant impact on participants’ beliefs about preventive behaviours, although the intervention was associated with more frequent use of moisturizing creams by ICU nurses. Possible reasons for the lack of effect include the low prevalence of severe dermatitis, participants’ high level of baseline beliefs about the importance of using hand moisturizers, and low uptake of the BCP. Face‐to‐face delivery of the intervention might have been more effective, but is unlikely to be cost‐effective.

Our bespoke method for assessing and grading dermatitis from photographs^23^ reduced the potential for subjective variation between observers, and allowed joint assessment by two experienced dermatologists in difficult cases. Moreover, the assessment was conducted blind to trial arm allocation or timing of photographs.

Errors may have occurred in the reporting of health beliefs and behaviours. If the participants reported what they perceived as desirable answers, this could have biased the effect estimates in favour of the intervention. However, little benefit was found. Lack of data on the extent to which participants completed all (or only part) of the BCP was a notable limitation of this study. The differential rates of recruitment across the clusters is likely to have decreased overall the power of the study.

Our finding that the intervention had little if any impact on the prevalence of dermatitis concurs with recently published randomized trials,^26^, ^27^, ^28^ but should not be construed as evidence against the efficacy of preventive measures including reduced hand washing and frequent use of moisturizing creams. Although changes in the prevalence of dermatitis were not statistically significant, they were in the direction that might have been expected. Caution is warranted when interpreting the findings, generalizability and potential benefits of the intervention, as the two study groups were specifically selected for high risk of hand dermatitis. It is possible that the BCP was ineffective in these high‐risk populations, but it might be effective in nurses who are at lower risk. The components of our intervention are supported by evidence, appear to have no adverse effects and are relatively inexpensive. Therefore, these principles should continue to underpin strategies for preventing hand dermatitis in nurses. Healthcare employers should provide nurses with ready access to hand creams and rubs, but BCPs of the type we tested add little to best practice, and should not be adopted without further supportive evidence.
